# The Therapeutic Effect of Intranasal Administration of Dexamethasone in Neuroinflammation Induced by Experimental Pulmonary Tuberculosis

**DOI:** 10.3390/ijms22115997

**Published:** 2021-06-01

**Authors:** Jacqueline V. Lara-Espinosa, María Fernanda Arce-Aceves, Dulce Mata-Espinosa, Jorge Barrios-Payán, Brenda Marquina-Castillo, Rogelio Hernández-Pando

**Affiliations:** Sección de Patología Experimental, Instituto Nacional de Ciencias Médicas y Nutrición Salvador Zubirán, Mexico City 14080, Mexico; jvle_29031991@comunidad.unam.mx (J.V.L.-E.); mariferarce@ciencias.unam.mx (M.F.A.-A.); dulmat@yahoo.com.mx (D.M.-E.); qcjbp77@yahoo.com.mx (J.B.-P.)

**Keywords:** tuberculosis, glucocorticoids, intranasal, dexamethasone, neuroinflammation

## Abstract

Tuberculosis (TB) is an important infectious disease and a public health problem. The organs most frequently affected by TB are the lungs; despite this, it has been reported that TB patients suffer from depression and anxiety, which have been attributed to social factors. In previous experimental work, we observed that the extensive pulmonary inflammation characteristic of TB with high cytokine production induces neuroinflammation, neuronal death and behavioral abnormalities in the absence of brain infection. The objective of the present work was to reduce this neuroinflammation and avoid the psycho-affective disorders showed during pulmonary TB. Glucocorticoids (GCs) are the first-line treatment for neuroinflammation; however, their systemic administration generates various side effects, mostly aggravating pulmonary TB due to immunosuppression of cellular immunity. Intranasal administration is a route that allows drugs to be released directly in the brain through the olfactory nerve, reducing their doses and side effects. In the present work, dexamethasone’s (DEX) intranasal administration was evaluated in TB BALB /c mice comparing three different doses (0.05, 0.25 and 2.5 mg/kg BW) on lung disease evolution, neuroinflammation and behavioral alterations. Low doses of dexamethasone significantly decreased neuroinflammation, improving behavioral status without aggravating lung disease.

## 1. Introduction

Inflammation is a response of vascular living tissues to injury [1]. The inflammatory response is the coordinated activation of signaling pathways in resident tissue cells and inflammatory cells recruited from the blood that control inflammatory mediators’ levels. [2]. There are two types of inflammatory responses, acute and chronic. Acute inflammation refers to the instantaneous or early response to an injurious agent and is a defensive response that paves the way for repairing the damaged site and consists on the leukocytic infiltration of predominantly polymorphonuclear cells (neutrophils) [1]. Chronic inflammation results from a persistent injurious agent and is characterized by a leukocyte infiltrate constituted by mononuclear cells (macrophages, lymphocytes, plasma cells) [1] that can lead to tissue damage [3].

For a long time, the central nervous system (CNS) was considered as an immune-privileged tissue, isolated from peripheral immune cells unable to cross the blood–brain barrier (BBB) under normal conditions [4]. However, recent data indicate that the CNS is immune-competent and actively cooperative with the peripheral immune system [4]. 

Immune activation in the CNS always involves microglia and astrocytes, which contribute to the homeostatic regulation of the brain tissue [5]. Microglia and astrocytes become activated in response to danger and induce an inflammatory response through pro-inflammatory cytokines, chemokines, secondary messengers, and reactive oxygen species (ROS) [6]. This response, named neuroinflammation, could also be beneficial by shielding the brain from pathogens and neurotoxic agents and promoting tissue repair processes [7]. Uncontrolled neuroinflammation can also cause tissue damage through high glial cell activation, BBB permeability, and peripheral immune cells infiltration [8]. These changes eventually cause increased pro-inflammatory intermediaries production inside the CNS, which are neurotoxic and induce neurodegeneration. Pathological neuroinflammation is a process that underlies multiple CNS disorders [8].

In diseases with an inflammatory setting, first-line medication has traditionally been agents that soothe inflammation, like glucocorticoids (GCs) and the nonsteroidal anti-inflammatory drugs (NSAIDs) [9]. The mechanism of NSAID action is to inhibit cyclooxygenase (COX) enzymes, which participate in the metabolism of arachidonic acid producing the pro-inflammatory mediators prostaglandins [9]. GCs have generally been used to treat inflammatory and immune diseases due to their anti-inflammatory and immunosuppressive effects [10,11]. Dexamethasone (DEX) is a synthetic glucocorticoid steroidal anti-inflammatory agent able to attenuate the early expression of pro-inflammatory cytokines produced by activated microglia/macrophages [12]. GCs are administered intravenously, intramuscularly, or orally, all routes that require high doses to achieve a therapeutic concentration within the CNS, with several adverse side effects, such as diabetes, glaucoma, and osteoporosis when patients receive high doses systemically or over a long time [13,14]. Inhaled GCs (IGCs) have less oral bioavailability and a greater lipophilicity, thus a greater efficacy with fewer side effects, furthermore IGCs are the most effective treatment in asthma because they control symptoms and prevent exacerbations [15]. However, high-dose IGCs may be associated with systemic side effects including osteoporosis, reduced growth velocity in children, skin thinning, cataracts, and glaucoma [16]. Therefore, a treatment strategy to reduce these systemic side effects is necessary. Intranasal delivery (IN) has been shown to give a route to direct drug transport along the olfactory and trigeminal nerves, allowing brain access for small and large molecules, and even stem cells in therapeutic concentrations, avoiding the side effects of systemic administration [14,17,18]. 

Tuberculosis (TB) is an infectious disease caused by *Mycobacterium tuberculosis* (*Mtb*). TB is currently one of the top ten causes of death worldwide and is the principal cause of death from a single bacterial infectious agent [19]. Bacillus Calmette–Guérin (BCG), a liveattenuated strain of *Mycobacterium bovis* [20], is the only licensed vaccine against TB, and is the most widely used vaccine in history [21]. However, despite billions of individuals having been vaccinated in the past century, TB continues to be a serious threat to global health [21]. Furthermore, countries with a high prevalence of TB, such as India, Pakistan, and China, have reported a strong correlation between TB, depression, and anxiety [22]. The prevalence of depression among TB patients ranges between 1.71% and 87.5% [23,24] and anxiety between 7.14% and 74% [25,26]. It is well known that cytokines can reach and are overproduced in the brain during peripheral inflammatory process by three different mechanisms: the neural, cellular, and humoral pathways [27,28,29]. Pro-inflammatory cytokines such as Interferon-gamma (IFNγ) and Tumor necrosis factor α (TNFα) might contribute to the development of depressive disorder by regulating neuronal excitability, synaptic transmission, synaptic plasticity, and neuronal survival [30,31]. These mechanisms generate brain inflammation, which induces depression by diverse pathophysiological processes, such as disturbing monoaminergic neurotransmission, oxidative injury, and hippocampal neuronal damage [32]. Peripheral inflammation generated in the lung by *Mtb* infection could induce CNS inflammation and neuropsychiatric disorders, such as depression and anxiety, in TB patients. 

We have recently demonstrated neuroinflammation and different neuropsychiatric abnormalities in an experimental model of progressive pulmonary TB without brain infection [33]. We hypothesize that the GCs intranasal administration could decrease the neuroinflammation in TB mice, and thereby the behavioral abnormalities. The present study aimed to evaluate the IN administration’s efficiency of three different doses of DEX (L/DEX (0.05 mg/kg BW), M/DEX (0.25 mg/kg BW) and H/DEX (2.5 mg/kg BW)) on lung disease evolution, neuroinflammation and behavioral alterations in a murine model of pulmonary TB. 

## 2. Results

### 2.1. The Effect of Intranasal Dexamethasone (DEX) Treatment on Survival and Bacilli Loads in Experimental Pulmonary Tuberculosis

**Figure 1:**
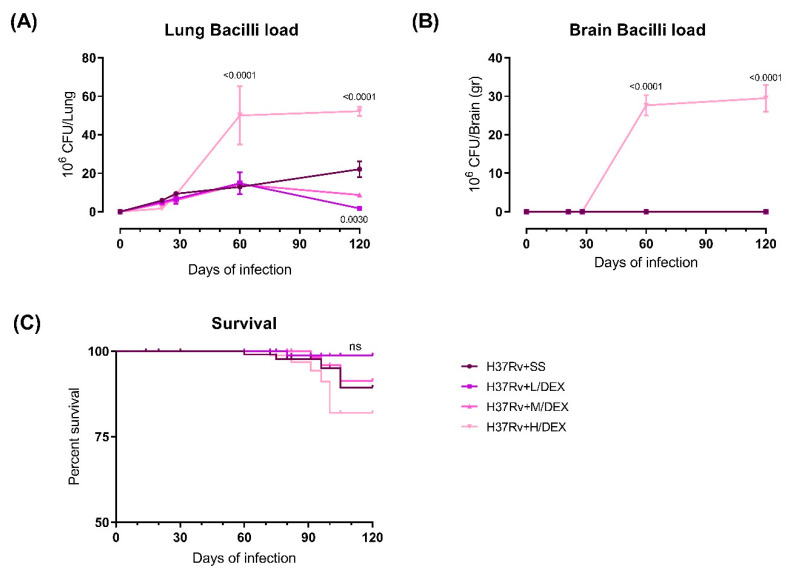
Effect of IN L/DEX (0.05 mg/kg BW), M/DEX (0.25 mg/kg BW) and H/DEX (2.5 mg/kg BW) starting in the early TB infection (14 days post-infection) on lung disease evolution of infected mice with 2.5 × 10^5^ colony-forming units (CFU) of *M. tb*. (**A**) Bacillary loads at lungs homogenates of control mice that only received the vehicle saline solution and infected animals treated with IN DEX F (3, 8) = 16.8, *p* = 0.0008, Two-way analysis of variance (ANOVA). Tukey’s multiple comparisons test. (**B**) Bacillary loads determined at brain homogenates from control mice and animals treated with saline solution and treated with IN DEX since day 14. F (3, 8) = 104.8, *p* < 0.0001, Two-way ANOVA. Tukey’s multiple comparisons test (*n* = 6). (**C**) Survival rates of control mice treated with saline solution and treated with IN DEX (*n* = 36). *p* = 0.2191, Logrank test for trend. Data are presented as mean +/− SEM. The H/DEX dose significantly increased the bacterial load in the lung and brain from day 60 post-infection and decrease survival rate, while the L/DEX and M/DEX doses did not increase the number of bacteria in these organs. Thus, bacterial load in the lung and brain from day 60 post-infection and decrease survival rate, while the L/DEX and M/DEX doses are safe to administer in animals infected with *Mtb*, as they do not aggravate lung disease.

Since a significant side effect of GCs administration in TB is the aggravation of the disease due to the suppression of cellular immunity, we first evaluated the effect of the IN administration of three doses of DEX (L/DEX (0.05 mg/kg BW), M/DEX (0.25 mg/kg BW) and H/DEX (2.5 mg/kg BW)), intending to find the dose that can reduce neuroinflammation without aggravating lung disease. We evaluated the effect of these treatments during the early and late phases of the disease, determining bacillary loads in the lungs and brains and the *M. tb*-infected animals’ survival. Infected mice treated with DEX by IN route since the early phase of infection (day 14 of infection) showed that the H/DEX dose significantly increased the lungs’ bacillary load on days 60 and 120 post-infection, while the M/DEX dose did not increase the pulmonary bacillary load and, interestingly, the L/DEX dose decreased the lung bacillary burdens compared to control animals on day 120 post-infection. In the brain only the H/DEX dose mycobacteria’s growth occurred on day 60 and 120 post-infection (Figure 1). The survival rate of animals that received IN H/DEX dose decreased compared to the control non-treated group (Figure 1). These results suggest that L/DEX and M/DEX doses do not aggravate the lung disease, while the H/DEX dose produced an aggravation of the lung disease when it was administered since the early stage of the infection. Therefore, L/DEX and M/DEX doses are safe to administer in animals infected with Mtb, and in the following experiments only L/DEX and M/DEX doses were used.

### 2.2. The Effect of Intranasal Low Dose DEX Treatment Since Early TB Infection on Diverse Behavioural Abnormalities 

**Figure 2:**
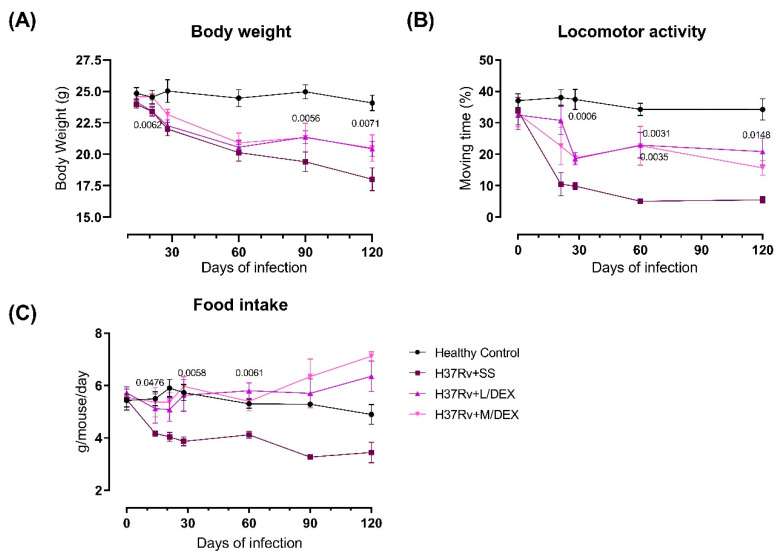
Effect of IN L/DEX (0.05 mg/kg BW) and M/DEX (0.25 mg/kg BW) in TB mice’s sickness behavior. (**A**) Bodyweight loss of infected animals that received L/DEX, M/DEX, control mice that only received the vehicle saline solution and healthy mice without infection. F (13, 117) = 5.316, *p* < 0.0001. Two Way ANOVA. Tukey’s multiple comparisons test (*n* = 6). (**B**) Locomotor activity of infected animals that received L/DEX, M/DEX, control mice that only received the vehicle saline solution and healthy mice without infection. F (2, 15) = 37.55, *p* < 0.0001. Two Way ANOVA. Tukey’s multiple comparisons test (*n* = 6). (**C**) Food intake of infected animals that received L/DEX, M/DEX, control mice that only received the vehicle saline solution and healthy mice without infection. F (3, 20) = 15.38, *p* < 0.0001. Two Way ANOVA. Tukey’s multiple comparisons test (*n* = 6). Data are presented as mean +/− SEM. The treatment with L/DEX and M/DEX since two weeks after infection decreased sickness behavior of infected animals. There is a decrease in the bacillary load on day 120 post-infection. The animals’ survival percentage improved. There was an improvement in body weight, an increase in locomotor activity and increase in food intake.

In previous work we observed that pulmonary infection with *M. tb* induced sickness behavior, manifested by a significant decrease in body weight and locomotor activity in infected animals [33]. Sickness behavior is a response related to the inflammatory process. Thus, we determined the effect of IN L/DEX and M/DEX doses administration during early Mtb infection on sickness behavior. The results showed that the treatment with L/DEX and M/DEX doses decrease the sickness behavior of TB mice (Figure 2). There was a slight improvement in body weight, mostly at 90- and 120-days post-infection and locomotor activity (LMA) was also significantly improved after one week of treatment. 

**Figure 3:**
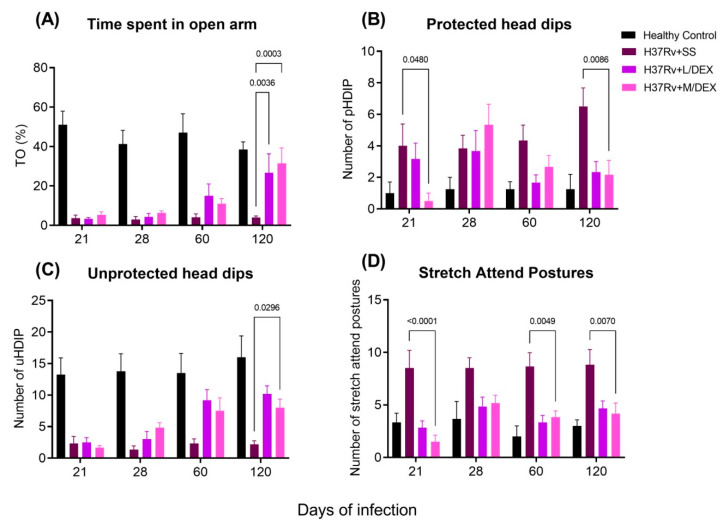
Effect of IN L/DEX (0.05 mg/kg BW) and M/DEX (0.25 mg/kg BW) on anxiety-like behavior in TB mice evaluated in the elevated I-maze. (**A**) Time spent by infected animals that received L/DEX, M/DEX, control mice that only received the vehicle saline solution and healthy mice without infection on the open arm (%TO). F (2, 15) = 7.571, *p* = 0.0053. Tukey’s multiple comparisons test (*n* = 6). (**B**) Protected head dips (Phdips) of infected animals that received L/DEX, M/DEX, control mice that only received the vehicle saline solution and healthy mice without infection. F (6, 45) = 2.449, *p* = 0.0391. Two Way ANOVA. Tukey’s multiple comparisons test (*n* = 6). (**C**) Unprotected head dips (uHDIPS) of infected animals that received L/DEX, M/DEX, control mice that only received the vehicle saline solution and healthy mice without infection. F (2, 15) = 11.47, *p* = 0.0009. Two Way ANOVA. Tukey’s multiple comparisons test (*n* = 6). (**D**) Stretched attend postures (SAP) of infected animals that received L/DEX, M/DEX, control mice that only received the vehicle saline solution and healthy mice without infection. F (2, 15) = 12.35, *p* = 0.0007. Two Way ANOVA. Tukey’s multiple comparisons test (*n* = 6). Data are presented as mean +/− SEM. DEX produced a significant increase in %TO, an increase in uHDIPS, a decrease in pHDIPS, and a decrease in SAP compared to the saline-treated mice at days 60 and 120 post-infection. All these data suggest that DEX has an anxiolytic-like activity on TB mice.

Another crucial behavioral change observed in both animals infected with Mtb and TB patients is anxiety. Indeed, we found that lung inflammation generated anxiety-like behaviour in our model of pulmonary TB [33]. Thus, we evaluated the IN L/DEX and M/DEX treatment’s effect on infected animals that showed anxiety-like behavior using the elevated I-maze [34], which is a modification of the elevated plus-maze model of anxiety in mice. We observed that the treatment with L/DEX and M/DEX doses increased the time spent by mice on the open arm (TO) and unprotected head dips (uHDIPS) at 60 and 120 post-infections in TB mice, and also reduced the protected head dips (pHDIPS) and stretched attend postures (SAP) (Figure 3). Thus, the treatment with IN DEX showed an anxiolytic-like activity on TB mice. 

**Figure 4:**
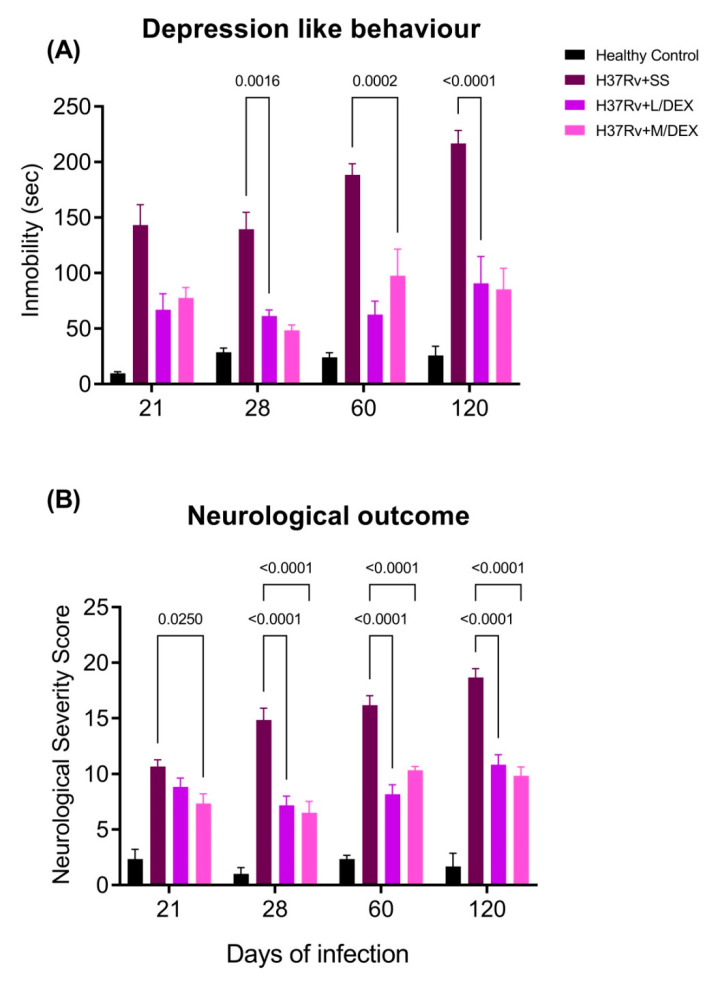
Effect of IN L/DEX (0.05 mg/kg BW) and M/DEX (0.25 mg/kg BW) in depression and neurological damage of TB mice. (**A**) Depression-like behavior of mice treated with IN DEX. F (2, 15) = 79.85, *p* < 0.0001. Two Way ANOVA. *p* < 0.0001. Tukey’s multiple comparisons test (*n* = 6). (**B**) Neurological outcome of mice treated with IN DEX. F (2, 15) = 53.69, *p* < 0.0001. Two Way ANOVA. Tukey’s multiple comparisons test (*n* = 6). Data are presented as mean +/− SEM. The treatment with DEX decreased depression-like behavior since day 21 post-infection and improved the neurological outcome of infected animals since day 28 post-infection.

We have previously demonstrated that pulmonary infection with *M. tb* induces depression-like behavior and neurological damage in a TB murine model [33]. We evaluated the IN DEX administration on these changes. The results showed that IN DEX decreased the depression-like behavior significantly in the Tail suspension test since day 21 post-infection (Figure 4). Similar results were seen in the evaluation of neurological damage, which significantly decreased since day 28 post-infection (Figure 4). It would seem that IN DEX had an anti-depressive effect and produced a beneficial effect on the neurological outcome of the infected animals. 

**Figure 5:**
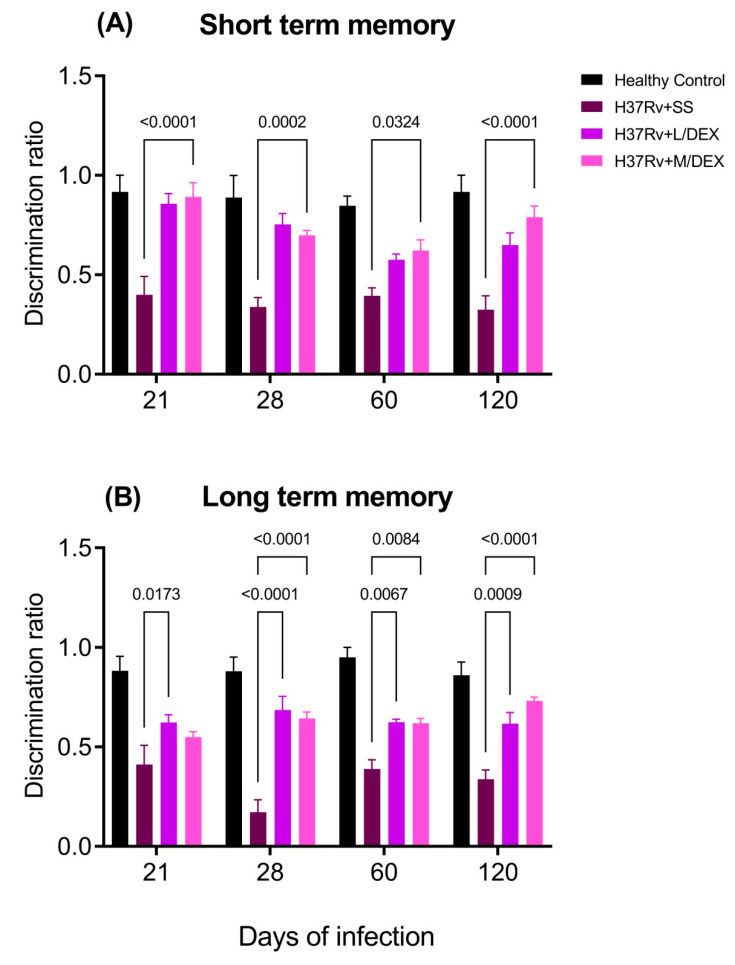
Effect of IN L/DEX (0.05 mg/kg BW) and M/DEX (0.25 mg/kg BW) on TB mice’s memory damage. (**A**) Short-term memory. F (2, 15) = 77.03, *p* < 0.0001. Two Way ANOVA. Tukey’s multiple comparisons test (*n* = 6). (**B**) Long-term memory. F (2, 15) = 50.69, Tukey’s multiple comparisons test (*n* = 6). Data are presented as mean +/− SEM. Animals with TB treated with IN DEX showed a significant improvement in short- and long-term memory.

Previously, we observed that pulmonary infection with *M. tb* induces damage in short-term memory from day 14 post-infection and in long-term memory from day 1 post-infection [33]. The IN DEX treatment improved the short- and long-term memory in the object recognition test of TB mice (Figure 5). 

### 2.3. The Effect of Intranasal DEX Treatment on Cytokine Expression in Different Brain Areas of TB Mice

**Figure 6:**
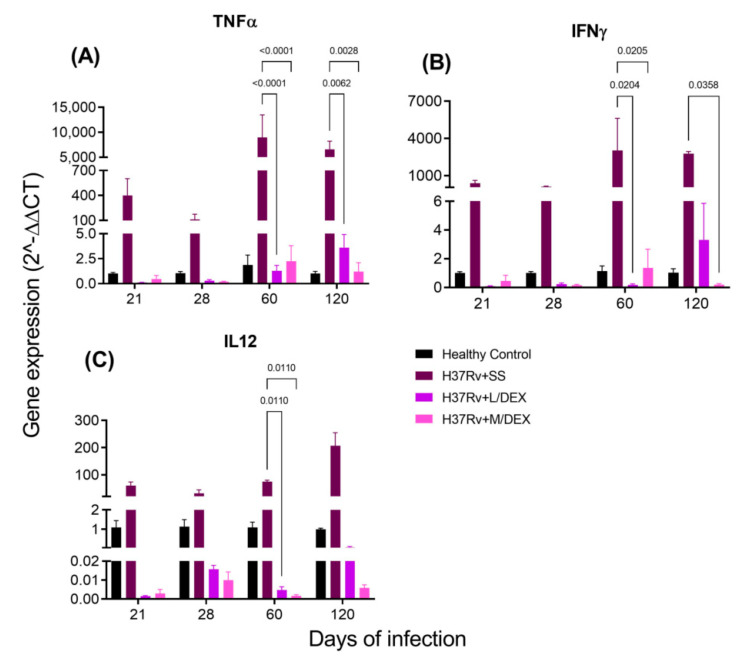
Effect of IN L/DEX (0.05 mg/kg BW) and M/DEX (0.25 mg/kg BW) on pro-inflammatory cytokines of the hippocampus of mice infected with *Mtb* H37Rv. (**A**) TNFα. F (3, 8) = 7.546, *p* = 0.0102. Mixed-effects model (REML). Dunnett’s multiple comparisons test. (**B**) IFNγ. F (3, 8) = 3.701, *p* = 0.0617. REML. Tukey’s multiple comparisons test (**C**) IL-12. F (3, 8) = 76.07. REML. Dunnett’s multiple comparisons test. There is a significant increase in gene expression in the absence of any detectable brain infection since day 21 post-infection. The treatment with L/DEX and M/DEX doses reduced the expression of these pro-inflammatory cytokines. RNA was isolated from hippocampus homogenates and reverse-transcribed to cDNA, then analyzed for gene expression changes of the indicated cytokine. Fold-change values were normalized to expression levels of the healthy controls (*n* = 6).

**Figure 7:**
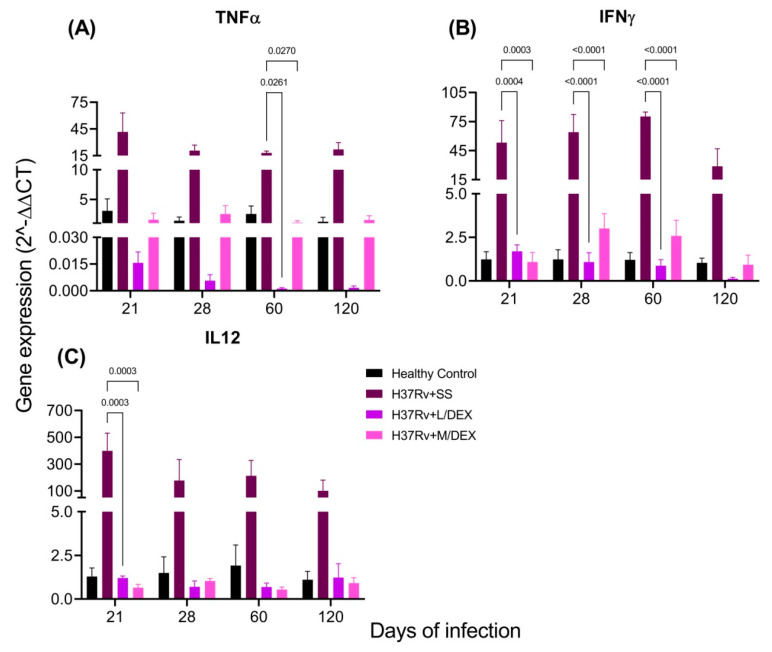
Effect of L/DEX (0.05 mg/kg BW) and M/DEX (0.25 mg/kg BW) on pro-inflammatory cytokines of the hypothalamus of mice infected with *Mtb* H37Rv. (**A**) TNFα. F (3, 29) = 14.50, *p* < 0.0001. REML. Dunnett’s multiple comparisons test. (**B**) IFNγ. F (3, 8) = 31.16, *p* < 0.0001. REML. Dunnett’s multiple comparisons test. (**C**) IL-12. F (3, 29) = 11.13, *p* < 0.0001. REML. Dunnett’s multiple comparisons test. There is a significant increase in gene expression in the absence of any detectable brain infection since day 21 post-infection. The treatment with L/DEX and M/DEX doses reduced the expression of these pro-inflammatory cytokines. RNA was isolated from hypothalamus homogenates, reverse-transcribed to cDNA, then analyzed for gene expression changes of the indicated cytokine. Fold-change values were normalized to expression levels of the healthy controls (*n* = 6).

**Figure 8:**
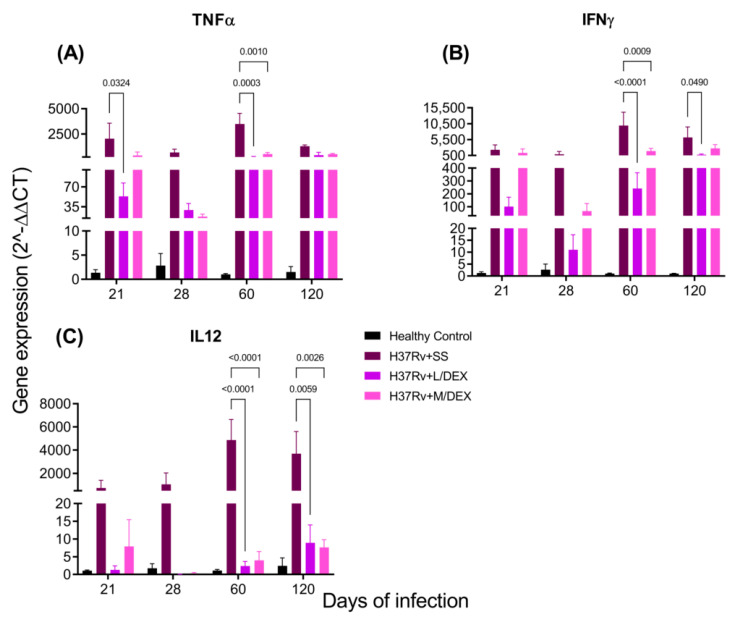
Effect of IN L/DEX (0.05 mg/kg BW) and M/DEX (0.25 mg/kg BW) on pro-inflammatory cytokines of the cerebellum of mice infected with *Mtb* H37Rv. (**A**) TNFα. F (3, 8) = 5.228, *p* = 0.0273. REML. Dunnett’s multiple comparisons test. (**B**) IFNγ. F (3, 8) = 6.175, *p* = 0.0177. REML. Dunnett’s multiple comparisons test. (**C**) IL-12. F (3, 29) = 15.06, *p* < 0.0001. REML. Dunnett’s multiple comparisons test. There is a significant increase in gene expression in the absence of any detectable brain infection since day 21 post-infection. The treatment with L/DEX and M/DEX doses reduced the expression of these pro-inflammatory cytokines. RNA was isolated from cerebellum homogenates, reverse-transcribed to cDNA and then analyzed for gene expression changes of the indicated cytokine. Fold-change values were normalized to expression levels of the healthy controls (*n* = 6).

**Figure 9:**
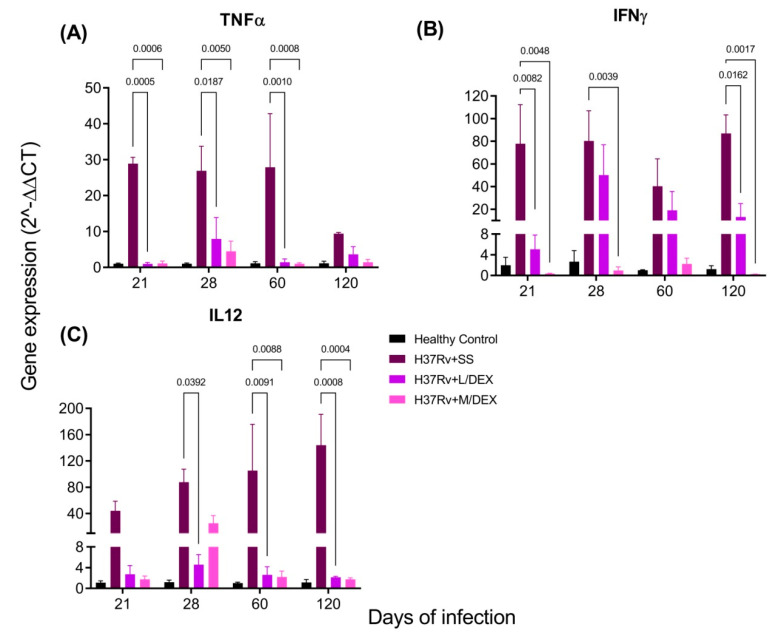
Effect of IN L/DEX (0.05 mg/kg BW) and M/DEX (0.25 mg/kg BW) on pro-inflammatory cytokines of the frontal cortex of mice infected with *Mtb* H37Rv. (**A**) TNFα. F (3, 30) = 20.56, *p* < 0.0001. REML. Dunnett’s multiple comparisons test. (**B**) IFNγ. F (3, 8) = 15.47, *p* = 0.0011. REML. Dunnett’s multiple comparisons test. (**C**) IL-12. F (3, 8) = 6.149, *p* = 0.0179. REML. Dunnett’s multiple comparisons test. There is a significant increase in gene expression in the absence of any detectable brain infection since day 21 post-infection. The treatment with M/DEX dose reduced the expression of these pro-inflammatory cytokines. RNA was isolated from frontal cortex homogenates, reverse-transcribed to cDNA and then analyzed for gene expression changes of the indicated cytokine. Fold-change values were normalized to expression levels of the healthy controls (*n* = 6).

Pro-inflammatory cytokines in the brain are produced during pulmonary TB without bacterial infection in the SNC [33]. We determined by RT-PCR the effect of IN DEX in the brain expression of TNFa, IFNg and IL-12 levels in the hippocampus, hypothalamus, cerebellum and frontal cortex. Our results showed that 0.05 and 0.25 mg/Kg BW of IN DEX administration significantly decreased the expression of these cytokines in the hippocampus (Figure 6) and hypothalamus (Figure 7) of mice infected with *M. tb*, compared to the group that received saline solution. In the cerebellum, we observed that although there was a decrease in these pro-inflammatory cytokines, the reduction was lesser than in the hippocampus and hypothalamus (Figure 8). In the frontal cortex, both doses of DEX significantly decreased the expression of these pro-inflammatory cytokines, although a more significant effect was observed with the dose of 0.25 mg/Kg BW (Figure 9).

Altogether, these results suggest that intranasal administration of L/DEX and M/DEX doses reduces neuroinflammation in the murine model of experimental pulmonary TB.

Taken together, our results demonstrate that intranasal administration of L/DEX and M/DEX in a mouse model of pulmonary TB does not aggravate lung disease. Furthermore, it effectively reduces the gene expression of TNFα, IFNγ and IL-12 in the hippocampus, hypothalamus, cerebellum and frontal cortex of infected animals. The sickness behavior, anxiety and depression-like behavior decreased, and there was an improvement in short- and long-term memory.

## 3. Discussion

TB is a significant cause of mortality in developing countries. The incidence of this condition is still increasing [19]. A generalized pro-inflammatory state is an early characteristic of TB and contributes to severe pathology. Furthermore, TB patients suffer common mental disorders such as depression and anxiety [22]. Previously, we have shown that in the absence of cultivable bacteria in the brain, *M. tb* induces neuroinflammation, neuronal damage, and behavioral abnormalities during pulmonary infection, manifested by high production of different cytokines, mainly TNFα, IFNγ, and IL12 [33]. This condition produced neuronal death, so it requires therapeutic approaches that modulate this pro-inflammatory response to avoid neuronal injury and behavioral abnormalities. From the therapeutic perspective, IN drug administration is advantageous for treating CNS disorders, as demonstrated in multiple studies [35]. IN GCs delivery has recently been proven to control experimental neuroinflammation induced by systemic lipopolysaccharide (LPS) injection in an experimental model of sepsis [17]. In that report, IN delivery of DEX effectively reduced the percentage of the Glial fibrillary acidic protein (GFAP) brain cells that were increased by LPS, reduced the presence of neutrophils in the brain and decreased Interleukin-6 (IL) expression [17]. Interestingly, the protective anti-inflammatory effects were less pronounced when DEX was delivered at the same dose through the intravenous (IV) route. Similar results have been observed in a 60-min middle cerebral artery occlusion stroke model. IN DEX reduced strikingly the ischemic brain tissue damage, reduced blood-brain barrier permeability, decreased mortality and improved the Neurological deficit (7-point neuroscore scale) and weight of animals [14]. Another study showed that intranasal administration of methylprednisolone to mice with experimental autoimmune encephalomyelitis (EAE) suppressed the neuroinflammatory response and reduced immune cell infiltration and demyelination, in a similarly way to intravenous administration [36]. Thus, these results suggest that IN GCs administration might offer a more effective and practical alternative than systemic administration to treat neuroinflammation in diverse diseases which coincides with our results. 

GCs are steroid hormones produced by the adrenal gland and regulated by the hypothalamus–pituitary–adrenal axis (HPA) and are the most used anti-inflammatory and immunomodulatory agents [10]. Their therapeutic value is enormous in a wide range of autoimmune/inflammatory diseases, and GCs have been widely used for treating autoimmune disorders, allergies, allograft rejection, neuroinflammation, and neoplastic diseases [10]. In this context, DEX, a potent anti-inflammatory synthetic GC, has been used to reduce inflammation in different neuroinflammatory conditions [14,17,37]. In the present study, we showed that treatment with L/DEX (0.05 mg/kg BW) and M/DEX (0.25 mg/kg BW) through the IN route in the murine pulmonary TB model significantly decreased neuroinflammation, improving behavioral status without aggravating lung disease.

We show that treatment since early infection (two weeks post-infection) with L/DEX and M/DEX by IN route improves survival in TB mice, while H/DEX (2.5 mg/kg BW) showed no survival benefit and increased bacilli load in the lung and dissemination with bacterial growth in the brain of mice. Interestingly L/DEX treatment was associated with a significantly lower lung bacilli load on day 120 post-infection. Similar results have been found during early sepsis in mice, where L/DEX treatment significantly improved survival compared with control mice, while treatment with higher DEX concentrations did not. Besides, L/DEX significantly reduced bacteremia [38]. In recent years, it has been observed that GCs reinforce the innate immune system and repress the adaptive immune system to help resolve inflammation and restore homeostasis [39]. GCs induce the expression of Toll-Like Receptor 2 (TLR2), NOD-like receptors family pyrin domain containing 3 (NLRP3) inflammasome and the purinergic P2Y2 receptor (P2Y2R), all of these receptors participate in innate immunity [40,41,42]. The type of exposure to glucocorticoids and the basal state of the immune system are essential factors influencing the effects of GCs [10]. The innate immune system is fundamental for the initial immune response upon infection [43], thus it could be possible that the decrease in lung bacilli lung in the murine model of TB is related to reinforcement of innate immunity mediated by L/DEX. 

As we had observed that pulmonary infection by *M. tb* induced behavioral abnormalities [33], we investigated the IN DEX administration effect on diverse behavioral abnormalities. Our results showed that the treatment with IN L/DEX and M/DEX decreased sickness behavior, induced an anxiolytic effect, reduced the depression-like behavior, produced a beneficial effect on the neurological outcome and improved the short and long term memory of the pulmonary infected TB mice. Active TB starts as a pulmonary exudative inflammatory process. Th1 lymphocytes mediate the protective adaptive immune response against TB with high production of IFN-γ and CD8+ T cytotoxic cells in animals and humans (Table 1) [44]. Pro-inflammatory cytokines such as IFNγ and TNFα might induce the development of depressive disorder by affecting neuronal excitability, synaptic transmission, synaptic plasticity, and neuronal survival. These abnormal activities are produced by brain inflammation, which induces depression by diverse pathophysiological processes, such as disturbing monoaminergic neurotransmission, induce oxidative injury, and hippocampal neuronal damage. Indeed, DEX has been used to reduce inflammation in diverse neuroinflammatory conditions [14,17]. Different studies demonstrated that the anti-inflammatory effects of glucocorticoids such as DEX were attributable to the reduced expression of IL1β, IL2, TGFβ, and TNFα [42]. Diverse evidence based on experimental studies demonstrated that GCs participate in the survival and death of neurons in both neurodegenerative and neuroprotective processes [43]. The binomial effect of GCs in the brain depends on the levels of GCs [43]. This coincides with our results, demonstrating that L/DEX and M/DEX effects on behavioral abnormalities are associated with a marked decrease in the inflammatory response. We found that mRNA expression levels of the inflammatory cytokines TNFα, IL12 and IFNγ were significantly downregulated in the treated group compared to the control non-treated TB group. It is known that low doses of GCs are more beneficial, preserving the physiological metabolism of the neurons and the HPA axis [43]. Interestingly, relatively low dose DEX administration (1 mg/kg, intraperitoneal injection) has been shown to attenuate inflammation and decrease ED1-positive cells and three markers of inflammatory activation of microglia/macrophage in murine models of traumatic brain injury (TBI) [12,45]. Similar results were found with hydrogel-mediated local delivery of dexamethasone that reduced neuroinflammation and improved functional motor recovery [46]. Another study reported that dexamethasone alone (0.025 mg/kg) and the co-administration of melatonin and dexamethasone 24 h after TBI improved locomotor function and brain injury [47]. Thereby, DEX has an efficient protective effect on neuroinflammation. 

Summarizing the results obtained in this experimental study demonstrated the effectiveness of low dose GC IN administration as a new therapy to control neuroinflammation in chronic infectious diseases, such as pulmonary TB. IN drug delivery is desirable because it is noninvasive, and can therapeutically target the brain, reducing systemic side effects [18,48]. It is important to highlight that IN L/DEX and M/DEX did not aggravate the lung disease, thereby IN DEX could be used as a co-adjuvant treatment in conventional chemotherapy against TB. 

## 4. Materials and Methods

### 4.1. Reagents 

The Middlebrook 7H9 and 7H10 media and the OADC (oleic acid, albumin, dextrose and catalase) were obtained from Becton-Dickinson (Detroit, MI, USA). The Rneasy^®^ Mini Kit for RNA extraction, the Omniscript^®^ Reverse Transcription Kit for obtaining complementary DNA and the QuantiTectTM SYBR^®^ for RT-PCR were obtained from Qiagen (Germantown, MD, USA). The primers of the analysed cytokines were obtained from InvitrogenTM Thermo Fisher Scientific (Waltham, MA, USA). The DEX was obtained from Sigma Aldrich (Zwijndrecht, The Netherlands).

### 4.2. Animals

A total of 384 adult male BALB/c mice of eight weeks old were obtained from the animal house facility of the National Institute of Medical Science and Nutrition Salvador Zubiran (INCMNSZ), Mexico. Mice were group-housed (*n* = 5/cage) and randomly divided into two groups: control (CT, *n* = 144) and infected (H37Rv, *n* = 240). All the animals were kept in an accredited animal holding facility maintained at a controlled temperature (23 ± 1 °C) and humidity (50 ± 20%) under a 12:12 h light- dark cycle (lights on at 07:00 h). Food and water were provided ad libitum. All the animal experiments were done according to the guidelines of the ARRIVE and Mexican Constitution law NOM 062–Z00-1999 and approval by the Ethical Committee for Experimentation in Animals of the INCMNSZ in Mexico, protocol number: PAT-1865-16/19-1.

### 4.3. The Experimental Model of Pulmonary TB

The murine model of progressive pulmonary TB was described previously [50,51]. Briefly, the reference *M. tb* strain H37Rv was cultured in 7H9 medium with OADC enrichment. Mid-log-phase cultures were used for all experiments. *M. tb* were counted and stored at −80 °C until use. Bacterial aliquots were thawed and pulse-sonicated to remove clumps. After mice infection, the bacterial inoculum’s remnant was plated to confirm the CFU’s number and viability administered to the animals. Male BALB/c mice, 8 weeks of age, were anaesthetized in a gas chamber using 0.1 mL of sevoflurane per mouse. A blunt stainless-steel cannula with a small ball in its terminal end was inserted through the mouth and directed to the trachea. The cannula’s proper intratracheal placement was verified by palpation of the small ball from the cannula rubbing the tracheal rings. Mice were infected through intratracheal instillation with 2.5 × 10^5^ live bacilli.

Mice were maintained in a vertical position until spontaneous recovery. A total of 244 infected mice were maintained in groups of five in cages fitted with micro-isolators in a P-3 biosecurity level facility.

### 4.4. Experimental Design

**Figure 10:**
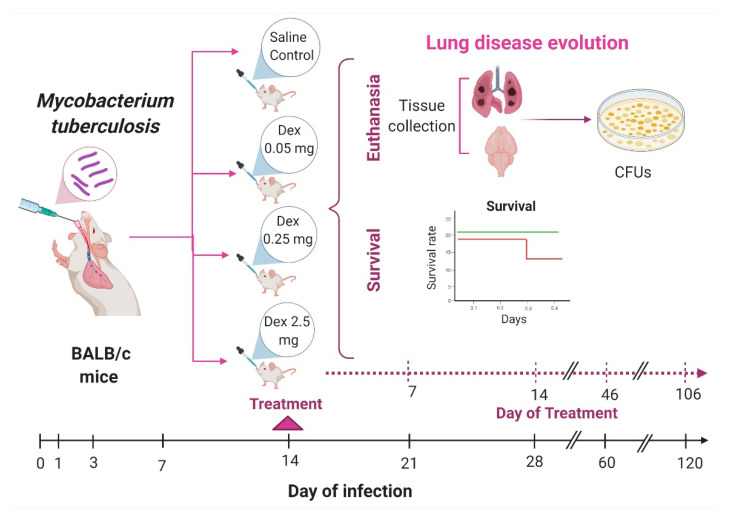
Study design workflow to evaluate the IN DEX treatment on lung disease evolution. BALB/c mice, 8 weeks of age, were infected with 2.5 × 10^5^ live bacilli. On day 14, the treatment with IN DEX started. We tried three different DEX doses: L/DEX (0.05 mg/kg BW), M/DEX (0.25 mg/kg BW) and H/DEX (2.5 mg/kg BW) by IN route; a control group received saline solution. On days 21, 28, 60, and 120 post-infection animals were euthanized, and the brain and lungs were collected to determine bacillary loads. During the complete experiment, we evaluated the survival of the animals. The samples for each experimental age group were run separately. (Created with BioRender.com, accessed on 20 May 2021).

**Figure 11:**
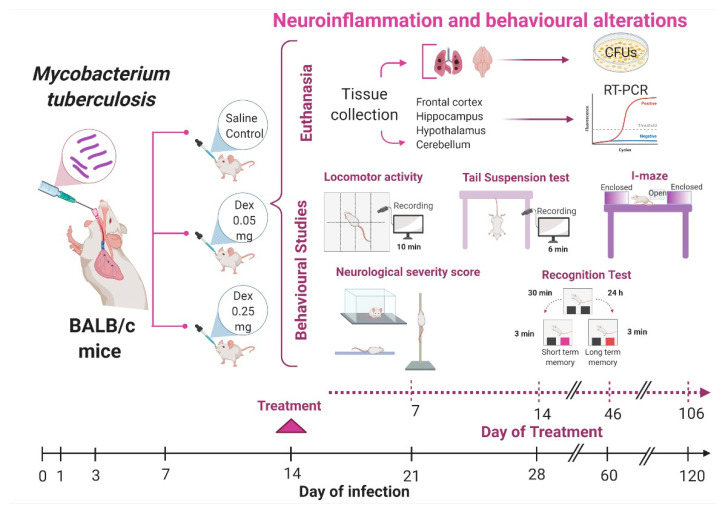
Study design workflow to evaluate the IN DEX treatment on neuroinflammation and behavioral alterations of TB mice. BALB/c mice, 8 weeks of age, were infected with 2.5 × 10^5^ live bacilli. On day 14, the treatment with IN DEX started. We used the L/DEX (0.05 mg/kg BW) and M/DEX (0.25 mg/kg BW) by IN route; a control group received saline solution. On days 21, 28, 60, and 120 post-infection, different behavioral tests were made. After the behavioral tests, animals were euthanized, and the brain and lungs were collected to determine bacillary loads. The hypothalamus, hippocampus, cerebellum, and frontal cortex were used to determine cytokines gene expression. For each of the measurements, two independent experiments were performed with *n* = 3 each. The samples for each experimental age group were run separately. (Created with BioRender.com, accessed on 20 May 2021).

We analyzed the effects of IN DEX administration on the CNS inflammation of pulmonary TB mice. In the first part of the work, we evaluated the effect of the treatment on lung disease (Figure 10). Following infection, mice were treated since day 14 post-infection and then euthanized by exsanguination under anesthesia at days 21, 28, 60, and 120 post-infection; lungs and brain were collected immediately to determine bacillary loads by CFU counts, and the survival was monitored during the complete experiment. In the second part of the work, we evaluated IN DEX administration’s effect on sickness behavior, behavioral abnormalities, and immune response in different brain structures (Figure 11). The selected areas of the brain (hypothalamus, hippocampus, cerebellum, frontal cortex) were immediately dissected by cutting with a razor blade, according to The Mouse Brain in Stereotaxic Coordinates [52]. The hippocampus was obtained underneath the frontal cortex, the cerebellum was identified as between the brainstem and the lateral recess of the 4th ventricle, the hypothalamus was obtained as the area lateral and medial to the fornix and the frontal cortex was obtained as the anterior part of the frontal lobes of the brain. Immediately after the dissection, the sample was frozen by immersion in liquid nitrogen and used to quantify cytokines gene expression by RT–PCR. Different behavioral tests were performed during pulmonary TB. These tests included the study of sickness behavior (LMA. Food intake and weight loss), anxiety-like behavior, NSS, short and long-term memory and depression-like behavior. Animals were monitored daily and were humanely euthanized under pentobarbital anesthesia if respiratory insufficiency, accentuated cachexia, or total immobilization was noted. Two independent experiments were performed.

### 4.5. Dexamethasone Administration 

After 14 days of infection, groups of three mice in two independent experiments were treated with 0.05 mg/kg, 0.25 mg/kg, or 2.5 mg/kg of dexamethasone administered by intranasal route (20 μL) three days per week (Monday, Wednesday, and Friday). Control mice received 20 μL of saline solution.

### 4.6. Determination of Colony-Forming Units (CFU) in Infected Lungs and Brain

Right lungs and the brains’ right hemisphere from six mice at each time point of two independent experiments were used for bacterial colony counting. Lungs and brains were homogenized with a FastPrep homogenizer (MP Biomedicals) in sterile tubes containing 1 mL of isotonic saline solution. Four homogenate dilutions were spread onto duplicate plates containing Bacto Middlebrook 7H10 agar, enriched with OADC. Incubation time and CFU counting were at 21 days of plating [51].

### 4.7. Expression of Cytokines by RT-PCR

Hippocampus, hypothalamus, cerebellums, and frontal cortex from six CT and infected animals at each time point were used to isolate mRNA using the Rneasy Mini Kit, according to the manufacturer’s recommendations. The quality and quantity of RNA were evaluated through spectrophotometry (260/280) and on agarose gels. Reverse transcription of the mRNA was performed using 100μg RNA, oligo dT, and the Omniscript kit. Real-time PCR was performed using the 7500 RT-PCR system (Applied Biosystems, Foster City, CA, USA) and Quantitec SYBR Green Mastermix kit (Qiagen). Negative controls were included in each PCR run. Specific primers for genes encoding glyceraldehyde-3-phosphate dehydrogenase (GAPDH) as housekeeping gene and for TNF-α, IFN-γ and Interleukin (IL) 12 were designed using the program Primer Express (Applied Biosystems, USA). Cycling conditions used were: initial denaturation at 95 °C for 15 min, followed by 40 cycles at 95 °C for 20 s, 60 °C for 20 s, and 72 °C for 34 s. Each sample was tested in duplicate. The fold change of gene expression was calculated by the 2^−(△△Ct)^ method [53].

### 4.8. Behaviour Tests

The behavior test methodology in the murine model of progressive pulmonary TB was described previously [33]. Briefly, animals were habituated to the test environment 24 h before it was made. Groups of mice were tested only once at the mentioned time points post-treatment to avoid potential habituation. All behavioral trials were performed during the first 4 h of the dark phase of the light cycle. The behaviors were analyzed and documented by a blind observer from these recordings. 

#### 4.8.1. Sickness Behavior 

To estimate sickness behavior, we evaluated LMA, food intake and weight loss. The effect of *M. tb* lung infection on LMA was evaluated in an open field by quantifying the mice’s moving time for 10 min. Data are represented as the per cent of moving during the 10 min. Twice a week the amount of food given to mice was weighed to determine food intake, and the total ingesting of food by mice was calculated. Data are expressed as g/mouse/day. The weight loss of the animals infected with *M. tb* was estimated from day one post-infection until day 120. Each week the animals were weighted, and their loss weight recorded. Data are represented as g of body weight. 

#### 4.8.2. Depression-Like Behavior

We evaluated depression-like behavior with the tail suspension test [54]. For this test, animals were suspended from the tail 6 min in a tripod 30 cm height, and their activity was recorded, focusing on the time that mice spent in behavioral despair. The time that the animal presented behavioral despair in those 6 min was recorded. 

#### 4.8.3. Anxiety-Like Behavior

The anxiety-like behavior was evaluated in the elevated I-maze, which modified the elevated plus-maze model of anxiety in mice [34]. The design of I-maze comprises a straight wooden passage, resembling the English letter “I,” divided equally into three areas; two enclosed areas (close arms) at both ends of the “maze” and an open area in the center of two enclosed areas. Animals were observed for 5 min duration, and %TO, Phdips, uHDIPS, and SAP were quantified.

#### 4.8.4. Neurological Outcome 

Motor function and reflexes of the infected mice were evaluated using a neurological severity score (NSS) [55]. They were valued regarding absent (0) or present (1), except for the hypomobility, motor impairment and balance that were rated as weak (1), moderate (2) or strong (3). The maximum score rates 31 (indicating neurological damage). Usual rate, from 3 to 6 (normal).

#### 4.8.5. Memory Damage 

Memory and learning after pulmonary infection with *M. tb* were assessed with the Object Recognition Test [56]. With this test, we evaluated short-term and long-term memory. In the first habituation phase, we placed the animal in the open field without any object for 10 min to become familiar with the environment. At 24 h, two identical objects (objects A) were placed in different positions, and the animal was left inside the box for 3 min. In the next phase, 30 min later, short-term memory was measured; for this, we positioned an object A (familiar object) and placed a new object in the other position (object B), the interactions with both objects (the animal sniffs or touches the object with the front legs) were counted during 3 min. After 24 h, the long-term memory was measured, for which object B was changed to a novel object (C), and the same procedure was followed. The results are presented as the discrimination ratio, which is the difference in interactions expressed as a proportion of the two objects’ total interactions in both tasks.

### 4.9. Statistical Analysis 

Data are expressed as the mean ± standard error of the mean (SEM) of two independent experiments. All data collection was randomized and tested with the Shapiro–Wilk normality test. The survival curves were analyzed with Logrank test for trend. Statistical significance of the bacilli load, body weight, locomotor activity and the behavioral tests was assessed using two-way ANOVA, followed by Tukey’s multiple comparisons test or Dunnett’s multiple comparisons test (comparison of each group against the saline control) as specified in the related text. The mixed-effects model analyzed cytokine expression by RT-PCR. Statistical significance was set at *p* < 0.05 for all experiments. Statistical analyses were performed in GraphPad Prism (v 9.1.1.225) (GraphPad, San Diego, CA, USA) [57]. 

## 5. Conclusions

**Figure 12:**
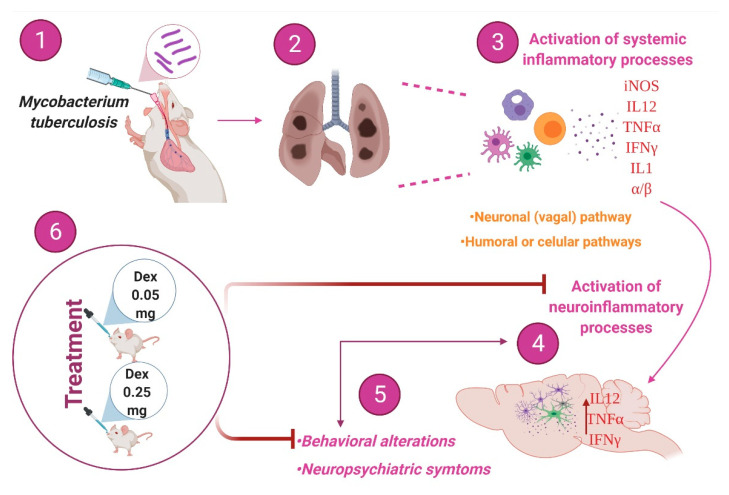
Intranasal administration of DEX decreases neuroinflammation and behavioral and neuropsychiatric symptoms in a murine model of pulmonary TB without aggravating lung disease. (**1**) The pulmonary infection with *M. tb* in a murine model (**2**) promotes the development of active disease in animals. (**3**) Intense inflammation due to the immune response against mycobacteria in the lungs induces neuroinflammation by humoral and neuronal pathways, (**4**) manifested by high production of different cytokines (**5**), induces behavioral alterations and neuropsychiatric symptoms such as depression and anxiety. (**6**) The intranasal administration of DEX reduces the production of cytokines due to the anti-inflammatory effect of this GC, and allows the use of low doses, being beneficial to the infected animals (Created with BioRender.com, accessed on 20 May 2021).

The intranasal administration of dexamethasone at low doses reduced TNFα, IFNγ, and IL12 expression in the hippocampus, cerebellum, hypothalamus, and frontal cortex of animals infected with *M. tb*. The treatment improved the behavioral changes present in animals with TB, decreased sickness behavior, depression, anxiety, neurological damage, and memory damage (Figure 12). It is important to note that it does not aggravate the lung disease so it could be used as adjuvant therapy to conventional anti-tuberculosis treatment. These are ongoing experiments in our laboratory. 

## Figures and Tables

**Table 1:** More representative cytokines affected by TB and the effect of DEX treatment ^1^.

Cytokine	TB	DEX
TNFα	Increases	Decreases
IFNγ	Increases	Decreases
IL12	Increases	Decreases
iNOS	Increases	Decreases
IL1β	Increases	Decreases
IL6	Increases	Decreases

^1^ DEX exerts an excellent inhibitory effect on inflammatory factors [49]. The systemic administration of DEX in TB would decrease the pro-inflammatory response, which is essential for protecting against the bacilli in this infectious disease. IN DEX allows the direct treatment to the CNS. Therefore, it does not aggravate lung disease.

## Data Availability

The data presented in this study are available on request from the corresponding author.

## Abbreviations

ANOVA	Analysis of variance
BBB	Blood-brain barrier
BCG	Bacillus Calmette–Guérin
BW	Body weight
CFU	Colony-forming units
CNS	Central Nervous System
CMD	Common mental disorders
DEX	Dexamethasone
EAE	Experimental autoimmune encephalomyelitis
GCs	Glucocorticoids
GFAP	Glial fibrillary acidic protein
H	High
HPA	Hypothalamus-pituitary-adrenal axis
IL	Interleukin
IGCs	Inhaled GCs
IN	Intranasal delivery
IFNγ	Interferon-gamma
IV	Intravenously
L	Low
LMA	Locomotor activity
LPS	Lipopolysaccharide
M	Medium
MCAO	Middle cerebral artery occlusion
MDRTB	Multidrug-resistant TB
*Mtb*	*Mycobacterium tuberculosis*
NSAIDs	Nonsteroidal anti-inflammatory drugs
OADC	Oleic acid, albumin, dextrose and catalase
pHDIPS	Protected head dips
REML	Mixed-effects model
ROS	Reactive oxygen species
SAP	Stretched attend postures
SEM	The standard error of the mean
TO	Time spent by mice on the open arm
TB	Tuberculosis
TNFα	Tumour necrosis factor-alpha
uHDIPS	Unprotected head dips
WHO	World Health Organization
